# Lower Antiplatelet Effect of Aspirin in Essential Thrombocythemia than in Coronary Artery Disease

**DOI:** 10.1055/s-0041-1731309

**Published:** 2021-07-04

**Authors:** Oliver Buchhave Pedersen, Anne-Mette Hvas, Hans Beier Ommen, Steen Dalby Kristensen, Erik Lerkevang Grove

**Affiliations:** 1 Thrombosis and Haemostasis Research Unit, Department of Clinical Biochemistry, Aarhus University Hospital, Aarhus, Denmark; 2 Department of Cardiology, Aarhus University Hospital, Aarhus, Denmark; 3 Department of Clinical Medicine, Faculty of Health, Aarhus University, Aarhus, Denmark; 4 Department of Hematology, Aarhus University Hospital, Aarhus, Denmark

**Keywords:** aspirin, essential thrombocythemia, platelet aggregation, thromboxane B2, platelet activation, platelet function test, coronary artery disease

## Abstract

**Background** Patients with essential thrombocythemia (ET) and coronary artery disease (CAD) have increased risk of thromboembolic complications. In addition, a reduced antiplatelet effect of aspirin has been demonstrated in both patient groups. As ET is a platelet disorder, platelets may be more important for the thromboembolic risk in ET than in CAD. We aimed to investigate the antiplatelet effect of aspirin and platelet turnover in ET versus CAD patients.

**Methods** We included 48 ET patients and an age-matched group of 48 CAD patients. The effect of aspirin was evaluated by thromboxane B _2_ (TXB _2_ ) levels and platelet aggregation. Platelet turnover was assessed by immature platelet count (IPC) and immature platelet fraction (IPF).

**Results** ET patients had reduced effect of aspirin compared with CAD patients, demonstrated by significantly higher TXB _2_ levels (median of differences = 22.3 ng/mL, *p* < 0.0001) and platelet aggregation (median of differences = 131.0 AU*min, *p* = 0.0003). Furthermore, ET patients had significantly higher IPC ( *p* < 0.0001) and IPF ( *p* = 0.0004) than CAD patients.

**Conclusion** ET patients have lower 24-hour antiplatelet effect of aspirin than CAD patients. This may be explained by an increased platelet production and turnover counteracting the antiplatelet effect of aspirin. These findings strengthen the rationale for exploring novel antiplatelet regimens in ET patients to reduce the risk of cardiovascular events.

## Introduction

Patients diagnosed with essential thrombocythemia (ET) or coronary artery disease (CAD) both receive aspirin due to an increased thromboembolic risk. 1 2 However, in both patient groups, a reduced effect of aspirin has been demonstrated. 3 4 5 6 7 It is currently not clear whether these patients' groups have different antiplatelet effect of aspirin and if so, whether differences in platelet turnover might contribute to the underlying mechanisms.

ET is a myeloproliferative disorder characterized by increased platelet count and turnover primarily due to high platelet generation. 8 9 The increased thromboembolic risk in ET cannot solely be explained by increased platelet count, since extreme thrombocythemia with counts higher than 1000 × 10 ^9^ /L has been associated with lower risk of thrombosis, 4 perhaps explained by acquired von Willebrand's disease. 10 Increased platelet turnover may be an important factor to explain the reduced effect of aspirin not only in ET patients but also in other patient groups. 3 8

An increased platelet turnover results in an increased amount of reticulated, immature platelets in the circulation. 11 Immature platelets counteract the irreversible inactivation of platelets by aspirin due to release of newly formed platelets unaffected by aspirin. 11 Notably, the effect of aspirin has been found to be reduced particularly at the end of the usual 24-hour dosing interval. 12 13 14 This has fostered an interest in exploring the effect of other dosing regimens of aspirin in ET patients. 15 16 Additionally, immature platelets are larger and suggested to be more reactive than mature platelets, likely explained by residual ribonucleic acid (RNA) content that provides the ability to produce prothrombotic proteins contributing to increased platelet activation. 11 17 18 19 20

We investigated ET patients matched with stable CAD patients to explore differences in aspirin response and differences in platelet turnover as a proof of that platelets may have different impact on thromboembolic risk in ET than in CAD. The differential effects of 75 mg aspirin daily at 24 and 1 hour after aspirin intake were evaluated by thromboxane B _2_ (TXB _2_ ) and platelet aggregation measurements. We hypothesized that (1) ET patients have lower effect from aspirin during 24 hours than CAD patients, and (2) ET patients have higher platelet turnover demonstrated by higher immature platelet count (IPC) and immature platelet fraction (IPF) than CAD patients.

## Materials and Methods

### Study Population and Design

The study was an observational cohort study on 48 ET patients compared with data on 48 age-matched CAD patients obtained from a previous publication. 12 ET patients were included at the Department of Hematology by the attending physicians. All ET patients were diagnosed based on the World Health Organization (WHO) criteria 21 and all matched CAD patients had angiographically documented stable CAD. All included patients were older than 18 years and treated with nonenteric coated aspirin mono antiplatelet therapy (75 mg once daily) for at least 1 month upon study enrolment. Patients were excluded if they received any antithrombotic therapy other than aspirin. The study was approved by The Central Denmark Region Committees in Biomedical Research Ethics (Reference number: 1–10–72–426–17) and by the Danish Data Protection Agency (Journal number: 1–16–02–916–17). Informed consent was obtained from all patients, and the study was conducted in accordance with the Helsinki-II Declaration.

### Blood Sampling

Patients had one blood sample obtained from an antecubital vein using a 21-gauge needle with minimum of stasis 1 hour after aspirin intake and another blood sample 24 hours after aspirin intake. Platelet count, red blood cell count, white blood cell count, platelet distribution width, mean platelet volume, IPC, and IPF were assessed in whole blood anticoagulated with ethylenediaminetetraacetic (Becton Dickinson Bioscience, California, United States) using an automated hematological analyzer (Sysmex XN-9000, Norderstedt, Germany). Plasma-creatinine was analyzed in lithium-heparin tubes (Becton Dickinson Bioscience) using Cobas 6000 (Roche, Basel, Switzerland).

### Thromboxane B _2_

Serum TXB _2_ was measured using an enzyme-linked immunosorbent assay according to manufacturer's instructions (Cayman Chemical, Ann Arbor, Michigan, United States). After exactly 1 hour of clotting at 37°C, serum was collected after centrifugation in 10 minutes at 2,600 g and stored at −80°C until analysis. All samples were measured in duplicate. Samples with results outside the standard curve were reanalyzed with appropriate dilutions. In addition, samples were reanalyzed if the variation in the duplicate measurements was above 20%.

### Platelet Aggregation

Hirudin tubes (Roche, Basel, Switzerland) were used to collect whole blood for multiple electrode aggregometry analysis of platelet aggregation. Samples rested for 30 minutes after collection and analyzed within 2 hours using the Multiplate Analyser (Roche, Basel, Switzerland). 22 To induce platelet aggregation, arachidonic acid (ASPItest 0.5 mM) was used as agonist. Platelet aggregation was quantified as area under the curve (AUC, aggregation units [AU] x minutes). Measurements were repeated if AUC by each of the two electrodes pairs varied more than 20% from mean.

### Statistics and Sample Size Calculation

The distribution of all data was evaluated by Q-Q plots and histograms. Continuous data were described as mean ± standard deviation when data were normally distributed and, if not, as median and interquartile range (IQR). Categorical data were described by percentages. Differences between ET patients and CAD patients were analyzed with an unpaired *t* -test for normally distributed data and with Mann–Whitney U test for nonnormally distributed data. Correlation analyses were performed with Spearman's ρ for data not following normal distribution. All tests of significance were two-tailed, with a probability value of *p* < 0.05. All statistics was performed in GraphPad Prism 6 (GraphPad Software Inc., La Jolla, California, United States).

The primary outcome was the difference in delta values (value 1 hour after aspirin intake minus the value 24 hours after aspirin intake) of TXB _2_ between the two patient groups. Using our previously published data, the mean delta value in TXB _2_ levels in ET patients was found to be 23 ng/mL. 23 From published data with CAD patients, the mean delta value in TXB _2_ levels was found to be 5 ng/mL. 12 With a standard deviation (sigma) of 27 ng/mL, a significance level (2α) at 0.05, and a power (1-β) of 90%, a total of 48 individuals were needed in each group.

## Results

In total, 48 ET patients were included in the study and matched by age with 48 CAD patients previously studied by our research group. 12 Table 1 shows the baseline characteristics of the two patient groups. A relatively low number of males were included in the ET group (42%) compared with the CAD group (83%). Furthermore, 8% of ET patients were current smokers compared with 27% of CAD patients.

**Table 1:** Baseline characteristics of the study population with 48 patients with essential thrombocythemia (ET) and 48 patients with coronary artery disease (CAD)

	ET patients	CAD patients	Reference interval
**Demographics**			
Age, y	64.5 (52.0–72.0)	63.0 (54.0–68.0)	
Male sex, *n* (%)	20 (41.7)	40 (83.3)	
Current smokers, *n* (%)	4 (8.3)	13 (27.1)	
Body mass index, kg m ^−2^	24.2 (21.4–26.8)	27.7 (25.1–28.7)	18.5–25.0 ^a^
Previously thromboembolic complications, *n* (%)	5 (10.4)	48 (100)	
**Mutation status**			
JAK2 mutation, *n* (%)	32 (66.7)		
CALR mutation, *n* (%)	12 (25.0)		
MPL mutation or triple negative, *n* (%)	4 (8.3)		
Time from diagnosis, y	6.1		
**Biochemistry**			
Hemoglobin, mmol/L	8.6 (8.1–9.0)	9.2 (8.7–9,5)	7.3–10.5 ^b^
Leucocyte count, × 10 ^9^ /L	6.0 (4.9–9.0)	6.3 (5.2–7.8)	3.5–10.0
Platelet count, × 10 ^9^ /L	512.5 (393.8–623.5)	230.5 (196.3–282.8)	145–400 ^b^
Mean platelet volume, fL	9.8 (9.1–10.5)	10.8 (10.3–11.5)	6.5–12.0 ^b^
Platelet large cell ratio, %	23.1 (18.7–29.4)	30.9 (27.3–37.8)	11.9–66.9
Platelet distribution width, fL	10.9 (9.8–12.8)	13.0 (11.8–15.0)	9.9–16.1 ^b^
High immature platelet fraction, %	1.2 (0.7–1.5)	0.7 (0.5–1.0)	0.1–2.7
Creatinine, µmol/L	66 (60.0–78.8)	73.5 (65.3–81.8)	45–105 ^b^

Values are medians (interquartile range [IQR]) unless otherwise indicated. JAK2, Janus kinase 2; CALR, calreticulin. ^a^ According to World Health Organization; ^b^ Reference interval is combined for men and female.

### Serum TXB _2_

The primary outcome was the difference in delta values of TXB _2_ levels between the two patient groups, as shown in Table 2 . ET patients had significantly higher delta levels of TXB _2_ levels than CAD patients, demonstrating reduced suppression of TXB _2_ . Median of differences in delta values of TXB _2_ levels between the two groups was 22.5 ng/mL, *p* < 0.0001.

**Table 2:** Platelet function, reflecting the antiplatelet effect of aspirin, analyzed by platelet aggregation and serum thromboxane B _2_ measurements ( *n* = 48 in both patient groups)

ET patients			CAD patients				
		Difference			Difference	Comparison a	*p* -Value ^b^
1h	24h	(24 vs. 1h)	1h	24h	(24 vs. 1h)	ET vs. CAD	
** Thromboxane B _2_** ; TXB _2_ , (ng/mL)							
6.4	32.3	26.2	1.5	5.1	3.7	22.5	< 0.0001
(3.1–14.4)	(14.2–64.0)	(10.1–48.0)	(0.8–3.6)	(3.8–8.3)	(2.6–6.0)		
**Platelet aggregation** ; AA, (AU*min)							
455	786	251	434	522	119.5	131	0.0003
(258–615)	(474–934)	(99–362)	(266–595)	(400–696)	(52–192)		

Abbreviations: AA, arachidonic acid (ASPItest); AU*min, aggregation units * min.; CAD, coronary artery disease; ET, essential thrombocythemia; TXB2, thromboxane B2.

Data are shown as median (interquartile range).

a Median of differences (CAD vs. ET); ^b^ Calculated using the Mann–Whitney U test.

We found significantly higher TXB _2_ levels 1 hour after aspirin intake in ET patients than in CAD patients (median: 6.4 [IQR: 3.1–14.4] vs. median: 1.5 [IQR: 0.85–3.8] ng/mL, *p* < 0.0001). In addition, ET patients had higher TXB _2_ levels 24 hours after aspirin intake than CAD patients (median: 32.3 [IQR: 14.2–64.0] vs. median: 5.1 [IQR: 3.8–9.6] ng/mL, *p* < 0.0001).

When dividing TXB _2_ values with platelet count to express TXB _2_ levels per unit of platelets, ET patients still had statistically significant higher levels when comparing delta values (median: 0.051 [IQR: 0.021–0.088] vs. median: 0.016 [IQR: 0.009–0.024], *p* < 0.0001) and 24 hours levels (median: 0.069 [IQR: 0.039–0.107] vs. median: 0.024 [IQR: 0.016–0.046], *p* < 0.0001). TXB _2_ levels 1 hour after aspirin intake showed the same tendency but did not reach statistical significance (median: 0.012 [IQR: 0.007–0.024] vs. median: 0.007 [IQR: 0.004–0.017], *p* < 0.078).

### Platelet Aggregation

The differences in platelet aggregation from 1 hour after intake of aspirin to 24 hours after intake of aspirin were compared between the two patient groups. We demonstrated a significantly higher difference in platelet aggregation during 24 hours in ET patients than in CAD patients, as shown in Table 2 . Median of differences in platelet aggregation was 131 AU*min, *p* = 0.0003.

We found no difference in platelet aggregation 1 hour after aspirin intake between the two patient-groups (median: 455 [IQR: 258–615] vs. median: 434 [IQR: 266–595] ng/mL, *p* = 0.68). ET patients had significantly higher platelet aggregation values 24 hours after aspirin intake than CAD patients (median: 785 [IQR: 474–934] vs. median: 522 [IQR: 400–696] ng/mL, *p* = 0.021).

**Fig. 1:**
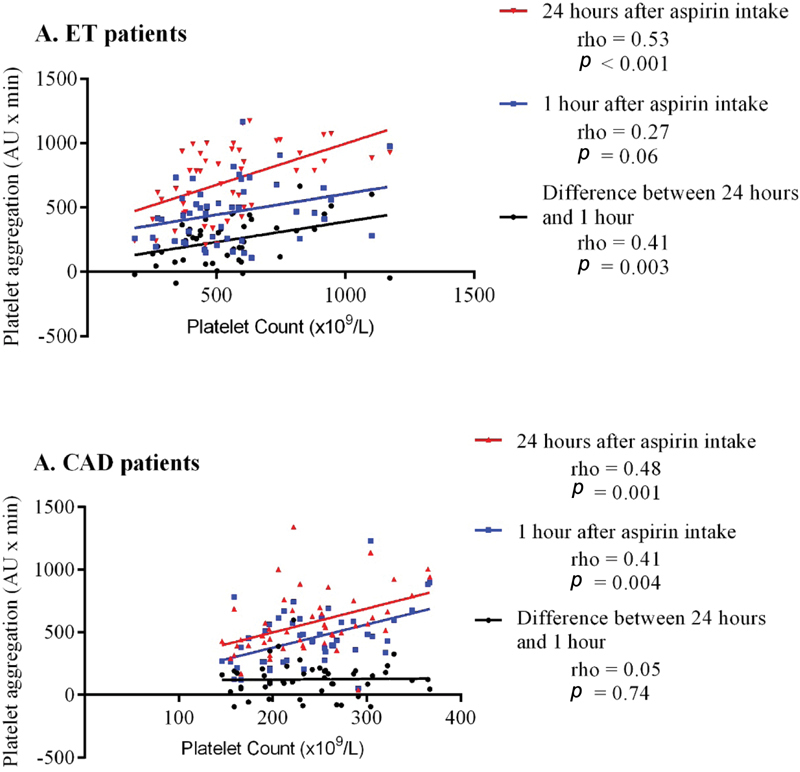
Correlation between platelet aggregation and platelet count: (1) 24 hours after aspirin intake, (2) 1 hour after aspirin intake, and (3) difference between 24 and 1 hour in **A** : 48 patients with essential thrombocythemia (ET) and **B** : 48 patients with coronary artery disease (CAD).

We attempted to correct platelet aggregation values for number of platelets. ET patients had significantly lower platelet aggregation 1 hour after aspirin intake (median: 0.88 [IQR: 0.53–1.21] vs. median: 1.74 [IQR: 1.29–2.44] ng/mL, *p* < 0.0001) and 24 hours after aspirin intake than CAD patients (median: 1.33 [IQR: 0.91–1.84] vs. median: 2.27 [IQR: 1.82–2.78] ng/mL, *p* < 0.0001) when dividing platelet aggregation values with platelet count. No difference was found in platelet aggregation during 24 hours between ET patients and CAD patients when dividing platelet aggregation values with platelet count. During 24 hours, platelet aggregation and platelet count were significantly correlated in ET patients (rho = 0.41, *p* = 0.003), but not in CAD patients (rho = 0.05, *p* = 0.74; Fig. 1) . At 24 hours following aspirin intake, platelet aggregation and platelet count were significantly correlated in ET patients (rho = 0.53, *p* < 0.001) and in CAD patients (rho = 0.48, *p* = 0.001; Fig. 1 ). At 1 hour following aspirin intake, platelet aggregation and platelet count were not significantly correlated in ET patients (rho = 0.27, *p* = 0.06), but significantly correlated in CAD patients (rho = 0.41, *p* = 0.004; Fig. 1 ).

### Platelet Turnover

**Fig. 2:**
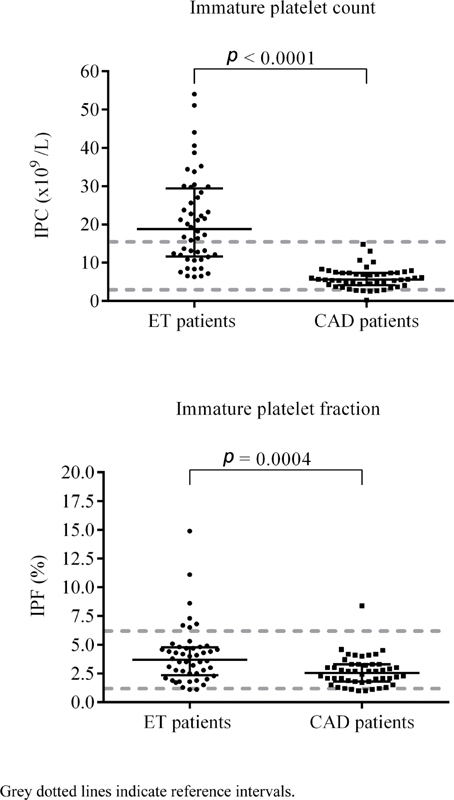
Comparison of immature platelet count (IPC) and immature platelet fraction (IPF) in 48 patients with essential thrombocythemia (ET) and 48 patients with coronary artery disease (CAD).

As demonstrated in Fig. 2 , IPC was significantly higher in ET patients than in CAD patients (median: 18.8 [IQR: 11.6–29.4] vs. median: 5.7 [IQR: 4.2–7.3] x10 ^9^ /L, *p* < 0.0001). Furthermore, IPF was also significantly higher in ET patients than in CAD patients (median: 3.7 [IQR: 2.4–4.8] vs. median: 2.6 [IQR: 1.8–3.3] x10 ^9^ /L, *p* = 0.0004).

**Fig. 3:**
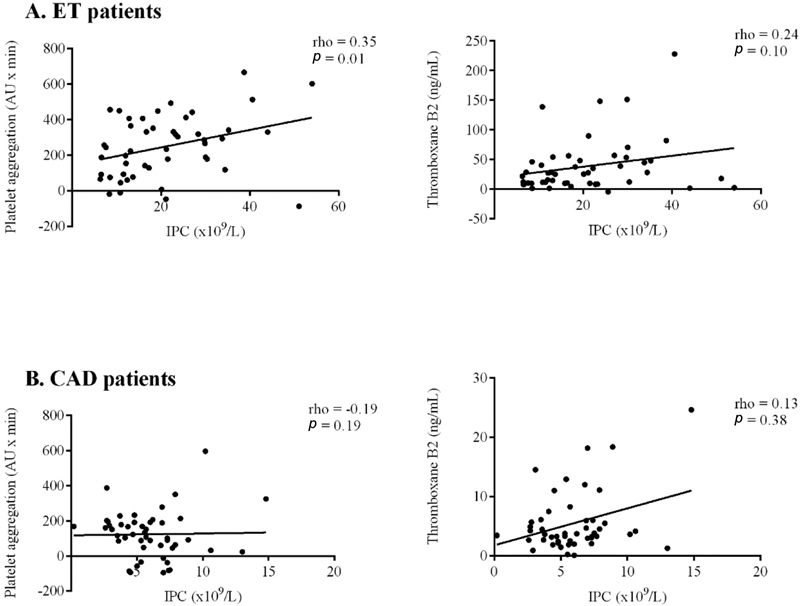
Correlation between immature platelet count (IPC) and (1) difference in platelet aggregation between 24 and 1 hour and (2) difference in thromboxane B _2_ levels between 24 and 1 hour in **A** : 48 patients with essential thrombocythemia (ET) and **B** : 48 patients with coronary artery disease (CAD).

As demonstrated in Fig. 3 **,** a statistically significant correlation between IPC and difference in platelet aggregation during 24 hours in ET patients was found (rho = 0.35, *p* = 0.01). No other significant correlations were observed between IPC and differences in TXB _2_ levels or platelet aggregation during 24 hours in ET or CAD patients ( Fig. 3) . No correlations between IPF and TXB _2_ levels or platelet aggregation (rho = − 0.19 to 0.24, *p* > 0.10) were found in ET or CAD patients.

## Discussion

We investigated the antiplatelet effect of aspirin in ET patients compared with stable CAD patients. Both patient groups have increased risk of thromboembolic complications and a reduced effect of once-daily aspirin monotherapy; however, the underlying mechanisms likely differ. Since ET is characterized as a platelet disorder, is it reasonable to assume that platelets may play a more important role in ET than in CAD for the risk of thromboembolic complications. The main finding of our study was that ET patients have significantly lower antiplatelet effect of aspirin during 24 hours than CAD patients. Furthermore, ET patients demonstrated higher platelet turnover, indicated by increased IPF and IPC compared with CAD patients.

We used two different methods to investigate the antiplatelet effect of aspirin: serum TXB _2_ measurements and platelet aggregation measured by impedance aggregometry. Both methods showed significantly lower antiplatelet effect of aspirin during 24 hours in ET patients compared with CAD patients. Determination of TXB _2_ levels is considered the most pharmacologically specific way to evaluate the antiplatelet effect of aspirin, because the contribution to its synthesis from other blood cells is minimal. 24 Additionally, dividing TXB _2_ measurements with platelet count had overall no influence on the results. Hence, TXB _2_ generation is inhibited to a lower extent throughout the day, indicating a lower antiplatelet response to aspirin treatment in ET patients than in CAD patients. A previous study demonstrated that increased platelet count contributed to increased residual TXB2 level prior to aspirin treatment, but not after aspirin antiplatelet treatment. 25 Platelet aggregation measured by impedance aggregometry is known to be positively associated with platelet count. 26 27 Indeed, when platelet aggregation results were divided by platelet count, the difference in antiplatelet effect of aspirin during 24 hours was no longer present indicating that the significantly higher aggregation found could, at least in part, be explained by the higher platelet count in ET patients. However, simply dividing platelet aggregation with platelet count is an imperfect way of adjusting for high platelet count, although studies found a linear relationship between platelet count up to 700 × 10 ^9^ /L and platelet aggregation. 26 27 28 29

Our finding of reduced effect of aspirin in ET patients is consistent with previous studies showing that monotherapy with low-dose aspirin results in insufficient platelet inhibition during the usual 24-hour dosing interval in ET patients. 3 23 This might be partly explained by an increased platelet count, as studies have shown that the effect of aspirin depends on the platelet count. 30 31 Furthermore, the type of aspirin may also be of importance, as plain aspirin may provide more efficient platelet inhibition than enteric-coated aspirin in patients with ET. 32 Aspirin exerts its effect by irreversible acetylation of the platelet COX resulting in inhibition of TXA _2_ biosynthesis lasting for an entire platelet life span. 33 Hence, the irreversible inhibition of aspirin compensates for the short half-life. 3 34 Under normal thrombopoietic conditions without increased platelet turnover, once daily aspirin provides sufficient platelet inhibition. 34 In addition, the irreversible inactivation of COX by aspirin also exerts its effect on the progenitor cells in the bone marrow, thus newly formed platelets are mainly nonfunctioning throughout the day. 35 36 37 However, patients with increased platelet turnover have a considerable release of platelets unaffected by aspirin into the bloodstream, 11 thus counteracting the irreversible COX inhibition. 11 In ET patients, studies have demonstrated an accelerated renewal of the drug target as a possible explanation for the reduced antiplatelet effect of aspirin. 3 5 38 A doubling of the aspirin dose given once daily may increase platelet inhibition immediately after administration, but still fails to provide sufficient 24-hour platelet inhibition. 3 39 Therefore, to obtain a more consistent platelet inhibition throughout the day, the dose interval should be decreased. 16 23 Accordingly, other studies on ET patients have shown that a twice-daily dosing of aspirin provides a more consistent platelet inhibition during 24 hours. 3 8 16 Furthermore, data from a large randomized controlled trial 16 indicate that twice-daily dosing of aspirin reduces the residual TXB _2_ level with around 90%, 40 whereas once-daily dosing of aspirin was insufficient in up to 80% of ET patients. 5 Notably, even a very low-dose of aspirin given twice daily (37.5mg) causes consistent platelet inhibition during 24 hours in ET patients. 23 In a new guideline, twice-daily aspirin treatment is now recommended for ET patients with arterial thrombosis if they are older than 65 years, have a Janus kinase 2 mutation, or have cardiovascular risk factors, as these factors have shown to be the major risk factors for thrombosis. 1 41 However, the ability of aspirin to prevent thromboembolic events in ET patients is still unclear and might be limited to only some subgroups of ET patients, 42 43 44 45 as low-dose aspirin in patients with very high platelet count may result in paradoxical occurrence of thrombosis and bleeding. 46 47 48 ET patients may also benefit from dual antiplatelet therapy with aspirin plus an adenosine diphosphate-inhibitor, 49 50 although only few studies have investigated this hypothesis.

The prothrombotic properties of immature platelets per se may also contribute to the reduced effect of aspirin. 11 19 20 Additionally, several studies have reported an association between increased levels of immature platelets and a high residual platelet activity evaluated by TXB _2_ measurements or platelet aggregation 18 51 52 In this study, IPC showed a statistically significant, though only moderately strong, positive correlation with platelet aggregation measured by impedance aggregometry in ET patients. No other significant correlations were observed between immature platelet markers and platelet function test in either of the patient groups. This is in accordance with previous studies, indicating that the absolute number rather than the relative fraction of immature platelets has a stronger correlation with platelet aggregation and TXB _2_ levels. 3 53 54 In addition, studies have demonstrated that especially ET patients with a history of thromboembolic complications have an increased platelet turnover compared with ET patients without thromboembolic complications. 55 56 Our study was not sufficiently powered to address this. Yet, it is still plausible that both the prothrombotic properties of immature platelets per se and an increased platelet turnover leading to the release of platelets unaffected by aspirin into the blood stream are concurrent mechanisms explaining our findings.

This study is the first study comparing strictly the WHO-defined ET patients with CAD patients to examine the importance of platelets to the antiplatelet effect of aspirin. We used two different methods to investigate the antiplatelet effect of aspirin, that is, serum TXB _2_ measurements and platelet aggregation. However, some limitations have to be considered. It should be acknowledged that CAD patients were originally included in a previous study. 12 As the two cohorts were only matched by age, they are likely to differ in other aspects. Different gender distribution was observed in the two groups, which may have influenced the results, as some studies have indicated that aspirin-treated women may more often have increased on-treatment platelet reactivity. 57 58 In addition, as smoking increases platelet aggregation, the differences in platelet aggregation observed between ET patients and CAD patients may be slightly underestimated as there were more smokers among CAD patients than ET patients. 59 We studied platelet aggregation in vitro, whereas the present study was not powered to assess clinical events. As we only included patients treated with aspirin monotherapy, our results may not be extrapolated to patients receiving other antithrombotic regimens.

## Conclusion

ET patients have reduced 24-hour antiplatelet effect of once-daily aspirin and an increased platelet turnover compared with CAD patients. Hence, platelets are possibly more important for the risk of thromboembolic complications in ET than in CAD. These findings strengthen the rationale for exploring novel antiplatelet drugs or aspirin dosing regimens 40 to overcome the reduced 24-hour antiplatelet effect of aspirin and reduce the risk of cardiovascular events.
